# Atypical Presentation of Tumor Lysis Syndrome Complicated by Rasburicase-Induced Methemoglobinemia

**DOI:** 10.7759/cureus.79823

**Published:** 2025-02-28

**Authors:** Mayisah Rahman, Fareeha Hussaini

**Affiliations:** 1 Medicine, Dr. Kiran C. Patel College of Osteopathic Medicine, Nova Southeastern University, Fort Lauderdale, USA; 2 Family Medicine, University of South Florida, Tampa, USA

**Keywords:** electrolyte disturbances, glucose-6-phosphate-dehydrogenase deficiency (g6pd), hyperuricemia, methemoglobinemia, rasburicase, tumor-lysis syndrome

## Abstract

Tumor lysis syndrome (TLS) is a life-threatening metabolic disorder caused by the rapid breakdown of malignant cells, usually associated with chemotherapy treatment. It can lead to electrolyte imbalances, such as hyperuricemia, hyperkalemia, hyperphosphatemia, and hypocalcemia. These disturbances can lead to complications, including acute renal failure, cardiac arrhythmias, and seizures. TLS typically presents in patients with hematologic malignancies; however, there has been an increase in cases in the context of solid tumors with comorbid conditions, bulky tumors, or dehydration. This report presents a case of TLS in an approximately 65-year-old female with stage IV squamous cell carcinoma of the cervix who developed acute kidney injury, lactic acidosis, and hyperuricemia following chemotherapy with docetaxel. Despite the absence of classic electrolyte abnormalities, her clinical decompensation raised suspicions of TLS. The patient was treated with rasburicase for hyperuricemia, from which she developed methemoglobinemia, a rare complication in patients with glucose-6-phosphate dehydrogenase (G6PD) deficiency. This case highlights the challenges in diagnosing atypical presentation of TLS in solid tumor patients. It also emphasizes the rare but serious complications of raburicase in G6PD-deficient individuals, such as methemoglobinemia. Further research into TLS in solid tumors and the role of G6PD screening in preventing adverse drug reactions in at-risk populations would be beneficial in these cases. Early recognition, rapid testing, and individualized treatment strategies are essential for patient care in these complex clinical scenarios.

## Introduction

Tumor lysis syndrome (TLS) is a metabolic disorder that occurs following the rapid destruction of malignant cells. This is usually associated with the initiation of chemotherapy and occasionally in non-malignant conditions that cause high cell turnover such as infections and rhabdomyolysis. This rapid cellular breakdown releases intracellular contents, including nucleic acids, potassium, phosphate, and uric acid, which can overwhelm the body’s excretory systems, leading to potentially life-threatening electrolyte imbalances. Acute kidney injury (AKI), severe metabolic acidosis, and hematuria may arise. Hyperuricemia, hyperkalemia, hyperphosphatemia, and hypocalcemia may lead to additional grave complications such as cardiac arrhythmias, seizures, and multi-organ failure [1].

Hyperuricemia, a hallmark of TLS, is concerning due to the risk of urate nephropathy, which can lead to acute renal failure through mechanisms of crystal precipitation and deposition, vasoconstriction, inflammation, and endothelial dysfunction caused by excessive histone release [2]. Management begins with the prevention of hyperuricemia through intravenous hydration, alkalinization with sodium bicarbonate, and urate-lowering therapy. Treatment often includes urate-lowering therapies such as allopurinol or a recombinant urate-oxidase enzyme that catalyzes the breakdown of uric acid to allantoin, known as rasburicase. While rasburicase is generally considered safe and effective, the drug can present with a rare complication when given to someone who is glucose-6-phosphate dehydrogenase (G6PD) enzyme deficient. This can induce methemoglobinemia as the drug oxidizes ferrous (Fe^2+^) to ferric (Fe^3+^) hemoglobin, impairing oxygen delivery to tissues despite an increased binding of oxygen to red blood cells [3]. This case report explores an atypical presentation of TLS superimposed with the clinical implications of rasburicase administration in a patient with previously unknown G6PD deficiency.

## Case presentation

An approximately 65-year-old African American female with a past medical history significant for recurrent stage IV cervical squamous cell carcinoma with recently diagnosed liver metastases on docetaxel presented to the hospital with dyspnea, lethargy, lower extremity swelling, and decreased appetite. Her symptoms of dyspnea began three weeks prior, seven days after initiating chemotherapy treatment. She additionally reported constant, non-radiating abdominal pain and fullness that began one month prior, which improved slightly with positional changes. She endorsed decreased appetite, orthopnea, bloating, increased urinary frequency, and darkening of urine.

**Figure 1:**
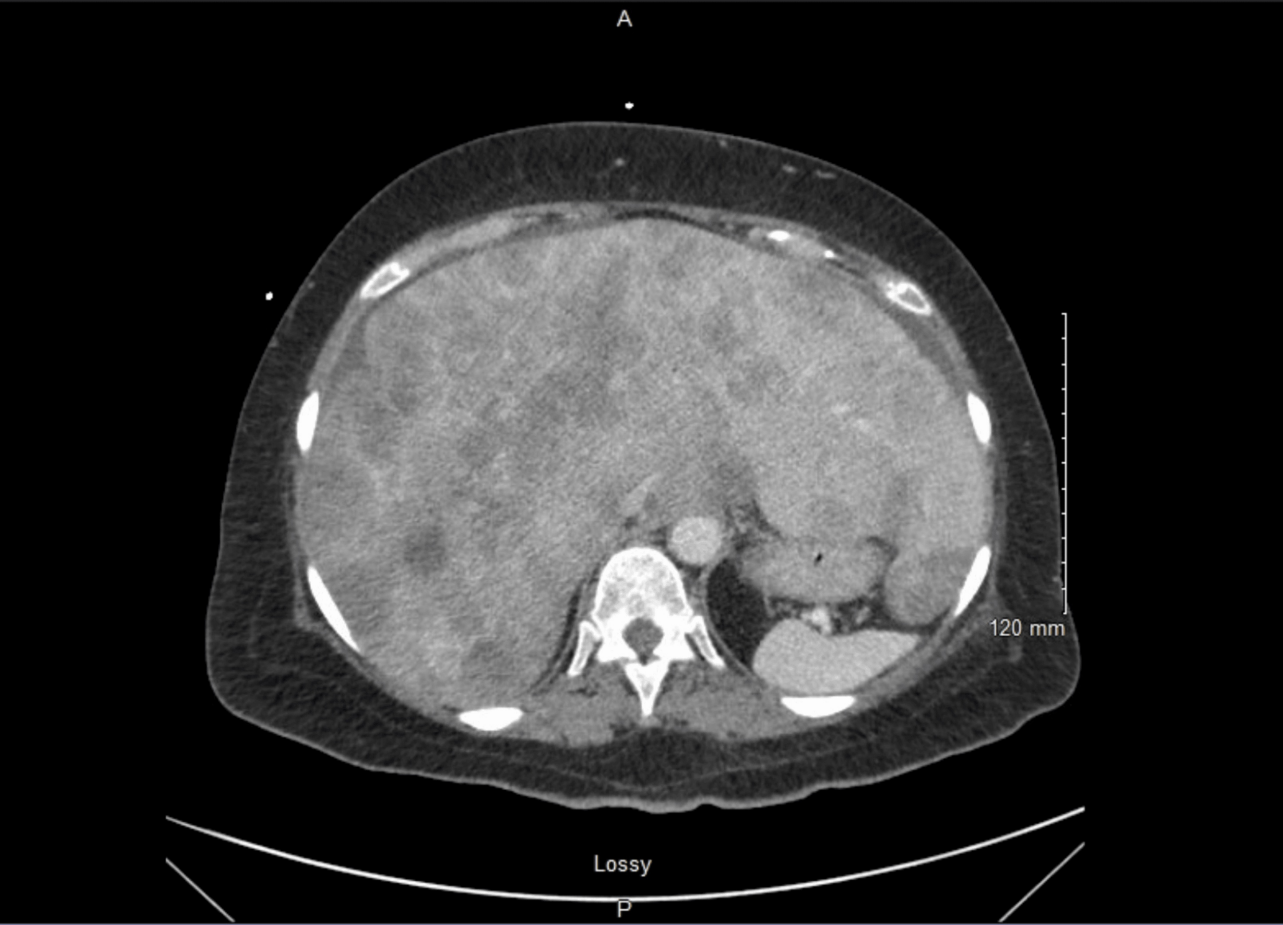
Axial view of CT abdomen and pelvis showing hepatic densities consistent with the patient’s history of stage IV cervical cancer with hepatic metastases and small-volume ascites.

**Figure 2:**
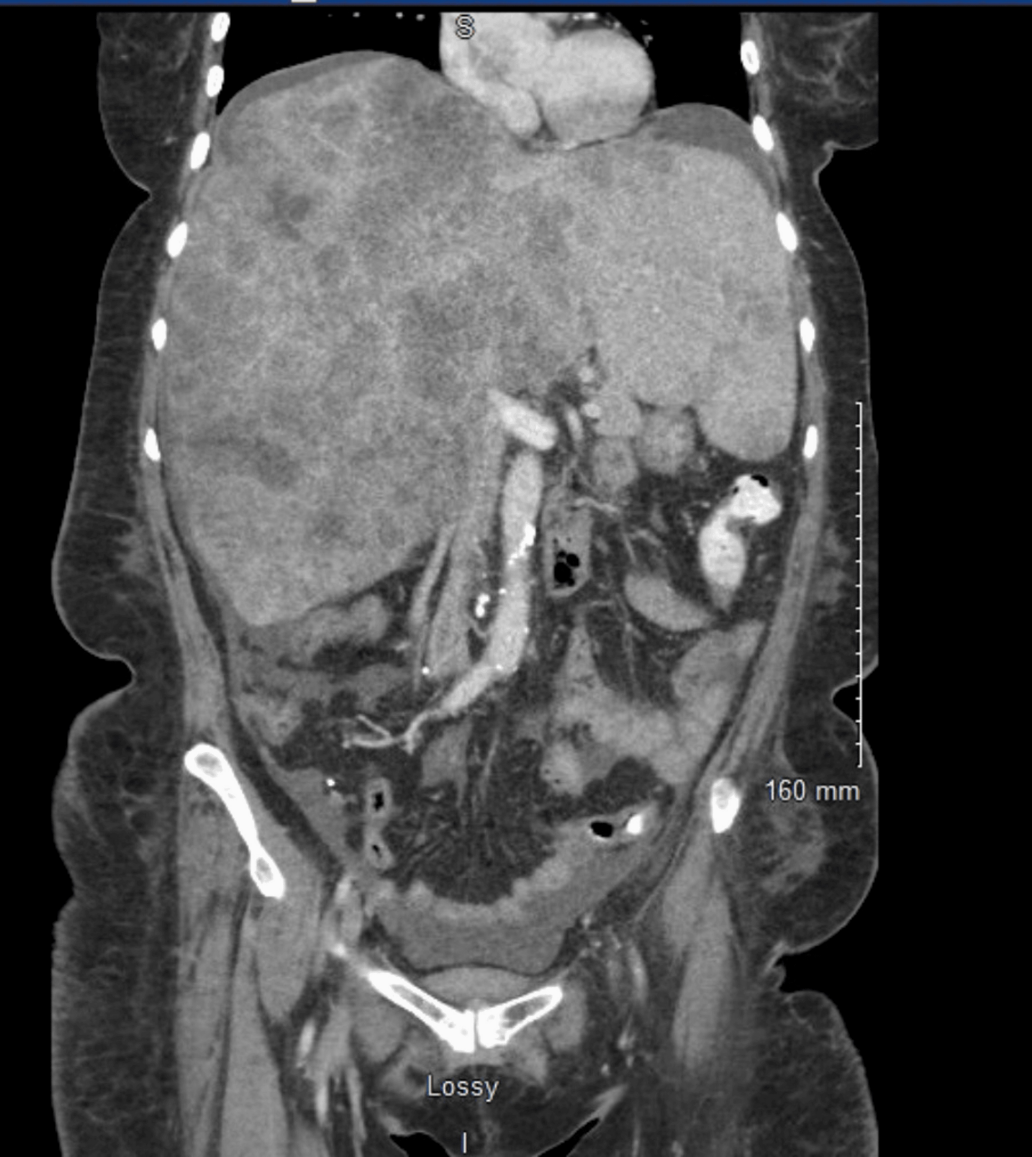
Coronal view of CT abdomen and pelvis showing hepatic densities consistent with the patient’s history of stage IV cervical cancer with hepatic metastases and small-volume ascites.

A physical exam revealed decreased breath sounds, abdominal tenderness to palpation in the right upper quadrant, and bilateral lower extremity edema. A CT angiogram was negative for pulmonary embolism and did not show any acute findings. CT abdomen and pelvis revealed known hepatic densities and small volume ascites (Figures 1-2).

Laboratory testing was significant for an AKI with creatinine levels two to three times the baseline, elevated C-reactive protein, transaminitis, mildly elevated white blood count, and lactic acidosis. Urinary analysis was also abnormal and questionable for a urinary tract infection (UTI) (Table 1).

**Table 1:** Lab values Bold values indicate results outside the reference range.

	Reference range	Patient values
Complete blood count (CBC)		
White blood cell count (WBC)	4.60-10.20 10^3/ uL	13.23
Red blood cell count (RBC)	4.04-5.48 10^6/uL	4.30
Hemoglobin (Hgb)	4.2-16.2 g/dL	13.3
Hematocrit (Hct)	37.7-47.9%	41.0
Platelet count	142-424 10^3/uL	245
Red cell distribution width (RDW)	11.6-14.6%	22.10
Nucleated red blood cells (NRBCs)	0.0-0.0/100 wbcs	0.60
Total nucleated red blood cells (total NRBCs)	0.00-0.00 10^3/uL	0.08
Comprehensive metabolic panel (CMP)		
Sodium	136-145 mmol/L	134
Potassium	3.5-5.1 mmol/L	3.9
Chloride	98-107 mmol/L	99
CO_2_	22-29 mEq/L	15
Blood urea nitrogen (BUN)	9.8-20.1 mg/dL	36.0
Calcium	8.40-10.20 mg/dL	10.60
Creatinine (Cr), blood	0.57-1.11 mg/dL	2.20
Aspartate aminotransferase (AST)	5.0-34.0 u/L	401.0
Alanine aminotransferase (ALT)	5-55 u/L	50
Total bilirubin	0.2-1.2 mg/dL	1.6
Alkaline phosphatase	40-150 u/L	217
Albumin	3.4-4.8 g/dL	2.9
Anion gap	5-13 mEq/L	20
BUN/creatinine ratio (BUN/Cr)		17
Glomerular filtration rate (GFR)	>=60	24
Lactate	0.5-2.2 mmol/L	3.6
Phosphorus	2.3-4.7 mg/dL	2.9
Complement C3	83-193 mg/dL	112
C4	15.0-57.0 mg/dL	32.0
Creatine phosphokinase (CPK)	29-168 u/L	91
Vitamin D	>30.0 ng/mL	34.3
Lactate dehydrogenase (LDH)	125-220 u/L	3782
Uric acid	2.6-6.0 mg/dL	20.8
C-reactive protein	0-0.5 mg/dL	4.28
Creatinine urine	47.0-110.0	163.4
Urinalysis (UA)		
Glucose	Negative mg/dL	Negative
Protein	Negative mg/dL	100
Urine urobilinogen	<2.0 mg/dL	1.0
Urine bilirubin	Negative	Small
pH		5.0
Urine ketones	Negative mg/dL	Trace
Urine Hgb	Negative	Large
Nitrites	Negative	Negative
Leukocytes	Negative	Large
Specific gravity	1.007-1.030	>1.030
Appearance	Clear	Turbid
Color	Yellow	Dark yellow
RBCs	0-2/hpf	>100
WBCs	0-5/hpf	>100
Squamous epithelial cells	0-5/hpf	3-5
Bacteria	/hpf	Few
Culture		10,000-100,000 cfu/mL *Escherichia coli*
PO2	35-45 mmHg	31
PCO2	35-45 mmHg	30
pH	7.35-7.45	7.36
Measured O2 Sat	>=70%	44
Base deficit	>= -2 mmol/L	-7
HCO3	24-27 mmol/L	17

Given her clinical presentation and objective findings, the differential diagnoses included AKI due to dehydration or nephrotoxic chemotherapy, new small-volume ascites from known liver metastases, urosepsis, and congestive heart failure (CHF). Although marked electrolyte abnormalities were not noted on the metabolic panel, clinical suspicion of TLS remained due to symptom onset after initiating chemotherapy, dyspnea, and labs indicating AKI.

She was maintained on IV hydration with normal saline with sodium bicarbonate and started on ceftriaxone for presumed UTI. Brain natriuretic peptide (BNP) levels were within normal limits, and the echocardiogram excluded the possibility of CHF. As her renal function worsened despite hydration, uric acid and lactate dehydrogenase (LDH) levels were ordered due to suspicion of TLS. Both results were significantly elevated, at 20.6 mg/dL and 3,868 U/L, respectively (Table 1). Due to her worsening dyspnea and AKI, a decision was made to start rasburicase for hyperuricemia while awaiting the G6PD enzyme test result. The following day, the patient developed persistent hypoxia with her pulse oxygen saturations (SpO_2_) ranging from 84% to 88% and did not respond to supplemental oxygen. The SpO_2_ did not correlate with the arterial blood gas (ABG) results, which showed 100% saturation. This discrepancy between pulse oximetry (SpO_2_) and arterial oxygen saturation (SaO_2_) is a classic finding in methemoglobinemia. A co-oximeter panel was ordered, demonstrating elevated methemoglobin levels at 9%, which resulted in immediate cessation of the medication, IV hydration, administration of ascorbic acid, and transfer to the intensive care unit for further management.

## Discussion

The present case demonstrates an atypical presentation of TLS in a patient with stage IV cervical squamous cell carcinoma, highlighting the challenges of diagnosing TLS in the absence of marked electrolyte abnormalities. The diagnostic challenges of TLS, compounded with the rare but serious complications associated with the use of rasburicase as treatment in a patient with high risk of G6PD deficiency, makes this situation worthy of further analysis.

While this case demonstrated the hallmark sign of hyperuricemia and clinical criteria of elevated creatinine, it lacked the hyperkalemia, hyperphosphatemia, and hypocalcemia [1] mentioned in the Cairo-Bishop criteria (Table 2). This absence of common signs made the diagnosis less obvious. However, the patient’s history of chemotherapy and worsening clinical condition despite hydration raised the alarm for TLS. The development of lactic acidosis, transaminitis, and abdominal pain likely caused by the carcinoma and metastases may also suggest systemic metabolic damage. This can occur even in the absence of severe electrolyte abnormalities. Ultimately, the significant elevation of uric acid (20.6 mg/dL) and LDH, acute renal failure, lower extremity edema, and clinical decompensation strengthened the suspicions [4].

**Table 2:** Cairo-Bishop criteria TLS: tumor lysis syndrome

Criteria	Laboratory diagnostic criteria (diagnosis requires the presence of two or more of the following)	Clinical diagnostic criteria (presence of laboratory TLS plus one or more of the following)
Uric acid	25% increase from baseline or ≥8.0 mg/dL	-
Potassium	25% increase from baseline or ≥6.0 mEq/L	-
Phosphorus	25% increase from baseline or ≥4.5 mg/dL (≥6.5 mg/dL in children)	-
Calcium	25% decrease from baseline or ≤7.0 mg/dL	-
Creatinine	-	Greater than 1.5 times the upper limit of normal of an age-adjusted reference range
Seizure	-	Presence of a seizure
Cardiac arrhythmia or sudden death	-	Presence of a cardiac arrhythmia or sudden death

TLS can be challenging to diagnose in patients who do not exhibit all the classic signs, particularly in those with solid tumors like this patient. This syndrome is more commonly associated with hematologic malignancies, such as acute leukemia, due to both its rapid growth rate and shorter time of effect [5]. Although acknowledged as less common, occurrence in solid tumors is becoming more recognized in the literature [6]. Several cases of TLS in solid tumors have shown an absence of the classic electrolyte abnormalities. Reviews report that hyperkalemia is absent in 28%, hyperphosphatemia in 17%, and hypocalcemia in 40% of cases [5]. Risk factors in the context of solid tumors correlate with this patient in regard to dehydration, bulky tumors, and rapidly growing tumors that spread to other organs. Early recognition, especially in cases of atypical presentation, is critical to prevent potentially fatal complications, including acute renal failure, cardiac arrhythmias, seizures, and possibly death [1].

Management of TLS begins with prophylactic strategies involving IV hydration to improve renal perfusion and filtration along with urate-lowering agents [4]. Given the compromised renal function in this case, rasburicase is the treatment method preferred over allopurinol and thus utilized in this patient. Rasburicase, a urate oxidase enzyme, has the advantage of rapidly reducing the amount of uric acid by catalyzing the conversion of uric acid to water-soluble allantoin. Although it is efficient in preventing AKI and managing TLS, it carries a rare but severe complication of methemoglobinemia. In G6PD-deficient individuals, oxidizing agents such as rasburicase can lead to the conversion of hemoglobin from its Fe^2+^ form to the Fe^3+^ form, producing methemoglobin [3]. The altered form of hemoglobin reduces the capacity to bind and release oxygen, leading to impaired tissue oxygen delivery. The oxidative stress can lead to hemolysis and potentially life-threatening anemia. Clinical manifestations may include symptoms of dyspnea, fatigue, and cyanosis. Objective findings involve decreased pulse SpO_2_ contradicting the normal partial pressure of oxygen (PaO_2_) on ABG [7]. The patient’s sudden drops in SpO_2_ were initially thought to be an error in pulse oximetry readings. However, after determining the hypoxia worsened after rasburicase administration, an ABG was ordered, revealing a normal PaO_2_. This prompted the suspicion of methemoglobinemia, as the main issue is not with the amount of oxygen in the plasma, but with the oxygen transport and delivery impaired by methemoglobin. The suspicion was confirmed by a co-oximeter panel demonstrating elevated methemoglobin levels. Although typically treated with methylene blue, clinicians must consider the possibility of G6PD deficiency. This is because methylene blue is contraindicated in patients with G6PD deficiency due to its ability to cause oxidative stress. In individuals with G6PD deficiency, there is a reduced capacity to regenerate nicotinamide adenine dinucleotide phosphate (NADPH), making the red blood cells more vulnerable to oxidative damage and hemolysis. Additional treatment options for methemoglobinemia include high-dose ascorbic acid. Thus, immediate cessation of rasburicase, administration of ascorbic acid, and appropriate management in the intensive care unit were the next critical steps in this scenario.

Populations at higher risk for this enzymatic deficiency include those of African, Mediterranean, or Southeast Asian descent [8]. Due to its X-linked inheritance, this condition is more common in males, leading to gaps in the literature regarding screening protocols of G6PD deficiency in females [9]. As the screening for G6PD deficiency can at times take up to a week to result, the risks and benefits are crucial to weigh prior to administration of rasburicase. Given that the complication rate is estimated to be below 1% [10], the benefits outweighed the rare risks, considering the severity of clinical decompensation.

The combination of these factors - an unexpected clinical presentation and an adverse drug reaction - underscores the importance of individualized treatment in the management of patients with malignancies undergoing chemotherapy. The increasing development of various therapies in solid tumors may alter the prevalence and presentation of TLS, but this remains an understudied area as it tends to be less frequently reported and under-recognized. Additional research is needed to better understand the frequency, risk factors, and presentation of TLS in solid tumor patients. Further studies are needed to establish early diagnostic markers for TLS that may not rely on the classic metabolic abnormalities. When considering management, there is limited guidance on the pre-treatment screening for G6PD deficiency in patients receiving rasburicase. Although routine screening for G6PD deficiency is not universally recommended, this case suggests that it should be considered in high-risk populations. Clinicians should also investigate the risks of heightened oxidative stress when rasburicase is being used. Broadening screening would be beneficial, but developing rapid testing for G6PD deficiency could prevent such scenarios, minimizing complications. More research is needed to determine whether routine screening and rapid testing for G6PD deficiency could prevent such complications. Further studies should explore whether alternative therapies may offer safer options for managing hyperuricemia in acutely decompensating patients when screening is unavailable.

## Conclusions

This case highlights the complexity of diagnosing and managing TLS in patients with solid tumors, especially in the context of an atypical clinical presentation. The case was further complicated by the rare complication of methemoglobinemia following rasburicase administration in a potentially G6PD-deficient patient. Clinicians must be aware of the full spectrum of TLS symptoms, the potential for atypical presentations, and the risks associated with commonly used treatments like rasburicase. Further research is needed to explore TLS in the context of solid tumors and identify alternative therapies when there are potential risks of contraindications. Rapid testing, early diagnosis, careful monitoring, and individualized treatment strategies are essential to improving outcomes in these high-risk patients.
